# Covert Treatment in Psychiatry: Do No Harm, True, But Also Dare to Care

**DOI:** 10.4103/0973-1229.40566

**Published:** 2008

**Authors:** Ajai R. Singh

**Affiliations:** * *Editor, MSM*

**Keywords:** Covert Treatment, Surreptitious prescribing, Beneficence, Non-malfeasance, Unhealthy staff practices, Autonomy, Secrecy, Malafide intent, Noncompliance, Relapse prevention, Insight-unconscious, Advance directive, Health care proxy, Dare to Care, Do no harm

## Abstract

Covert treatment raises a number of ethical and practical issues in psychiatry. Viewpoints differ from the standpoint of psychiatrists, caregivers, ethicists, lawyers, neighbours, human rights activists and patients. There is little systematic research data on its use but it is quite certain that there is relatively widespread use. The veil of secrecy around the procedure is due to fear of professional censure. Whenever there is a veil of secrecy around anything, which is aided and abetted by vociferous opposition from some sections of society, the result is one of two: 1) either the activity goes underground or 2) it is reluctantly discarded, although most of those who used it earlier knew it was needed. Covert treatment has the dubious distinction of suffering both such secrecy and disapproval.

Covert treatment has a number of advantages and disadvantages in psychotic disorders. The advantages are that it helps solve practical clinical problems; prevents delays in starting treatment, which is associated with clinical risks and substantial costs; prevents risk of self-destructive behaviour and/or physical assault by patient; prevents relapse; and prevents demoralization of staff. The disadvantages are that it maybe used with malafide intent by caregivers with or without the complicity of psychiatrists; it may be used to force conformity in dissenters; and the clinician may land himself in legal tangles even with its legitimate use. In addition, it may prevent insight, encourage denial, promote unhealthy practices in the treating staff and prevent understanding of why noncompliance occurs in the first place.

Some support its use in dementia and learning disorders but oppose it in schizophrenia. The main reason is that uncooperative patients of schizophrenia (and related psychoses) are considered to be those who refuse treatment but retain capacity; while in dementia and severe learning disorder, uncooperative patients are those who lack capacity. This paper disputes this contention by arguing that although uncooperative patients of schizophrenia (and related psychoses) apparently retain capacity, it is limited, in fact distorted, since they lack insight. It presents the concept of insight-unconsciousness in a patient of psychosis. Just as an unconscious patient has to be given covert medical/surgical treatment, similarly an insight-unconscious patient with one of the different psychoses (in the acute phase or otherwise) may also have to be given covert treatment till he regains at least partial insight. It helps control psychotic symptoms and assists the patient in regaining enough insight to realize he needs treatment. Another argument against covert treatment is that people with schizophrenia have the capacity to learn and therefore can learn that they are required to take medications, but if medications are given covertly it may well fuel their paranoia. However, it should be noted that the patient who has lack of insight cannot learn unless he regains that insight, and he may need covert treatment to facilitate this process. Covert treatment can fuel the paranoia, true, but it can also control the psychotic symptoms sufficiently so that regular treatment can be initiated. In a patient who refuses to accept that he is sick and when involuntary commitment is not an option to be considered, covert treatment is the only option, apart from physical restraint. Ultimately, a choice has to be made between a larger beneficence (control of symptoms and start of therapy) and a smaller malevolence (necessary therapy, but without the patient's knowledge and consent).

A number of practical clinical scenarios are outlined wherein the psychiatrist should adopt covert treatment in the best interests of the patient. Ethical issues of autonomy, power, secrecy and malafide intent arise; each of these can be countered only by non-malfeasance (above all, do no harm) under the overarch of beneficence (even above that, dare to care). An advance directive with health care proxy that sanctions covert treatment is presented. Questions raised by the practical clinical scenarios are then answered.

The conclusions are as follows: covert treatment, i.e, temporary treatment without knowledge and consent, is seldom needed or justified. But, where needed, it remains an essential weapon in the psychiatrist's armamentarium: to be used cautiously but without guilt or fear of censure. However, the psychiatrist must use it very judiciously, in the rarest of rare cases, provided: i) he is firmly convinced that it is needed for the welfare of the patient; ii) it is the only option available to tide over a crisis; iii) continuing efforts are made to try and get the patient into regular psychiatric care; iv) the psychiatrist makes it clear that its use is only as a stop-gap; v) he is always alert to the chances of malevolence inherent in such a process and keeps away from conniving or associating with anything even remotely suspicious; and vi) he takes due precautions to ensure that he does not land into legal tangles later.

The need of the hour is to explore in greater detail the need and justification for covert treatment, to lay out clear and firm parameters for its legitimate use, follow it up with standard literature and, finally, to establish clinical practice guidelines by unconflicted authors.

The term “covert treatment” is preferable to “surreptitious prescribing”; they should not be used synonymously, the latter term being reserved for those cases where there is malafide intent.

## I. Introduction; The Problem; Ethical Issues Involved; Where Justified, Where Not; Some Familiar Scenarios

### I.1. Introduction

The clinical psychiatrist is sometimes faced with a difficult situation: A patient's relative lands up in the clinic. [That's not the difficult part; why he lands up, is]. The problem is that the patient is unwilling to come for treatment. From the history given, the psychiatrist arrives at a tentative diagnosis that it is some form of psychosis. The relative is extremely distressed because of the patient's disorganized behaviour. He is also helpless because the patient just will not come for consultation. Most importantly, the relative appears genuinely interested in getting the patient treated. The psychiatrist knows that covert medication and empathetic handling can make the patient sufficiently calm so that he can be brought for treatment later. Should he or shouldn't he?

In all such cases, as we will see later, opinions are widely divided. In fact, one of the hugely contentious issues in psychiatry is the treatment of the uncooperative patient. In this category, the topic that arouses maximum debate, often bordering on acrimony, is covert treatment of an uncooperative patient. This actually means treatment without knowledge and/or consent of an individual considered a patient. It is often also called “surreptitious” treatment or prescribing, which terminology I shall avoid for reasons I will discuss later in this article.

Whenever a topic arouses heated debate, at this does, it means the issues are far from resolved and no clear-cut, universally approved guidelines or lines of action exist.

The opinion on the use of covert treatment in such a situation is likely to differ depending on who views it. A clinical psychiatrist who has handled such problems successfully might say, of course it should be used. But a psychiatrist who has been dragged to court for helping some such patient's relative earlier or one who knows a colleague who was similarly harassed, might advice against it. A neighbour who has had to bear the brunt of the anger, the assault and the shouting of such a patient may want involuntary hospitalization or whatever it takes to get “the fellow out of his neighbourhood,” or at least sufficiently controlled by covert medication or any other means so that the patient did not harass or hurt him. A human rights activist might see human rights infringement issues, and an ethicist might see profound questions of autonomy, beneficence, non-malfeasance, justice and breach of trust. However, a relative who has had to face such a problem in his household might plead with all: “enough of your discussions and objections, will someone offer me a solution on how to help my son/wife/husband/brother/father who locks himself up and believes the whole world is out to get him?” A lawyer may see a potential client in the patient given covert treatment. A law enforcement agency, like the police or the court, may smell a conspiracy to label and commit someone with intent to usurp property, gain separation, etc. And, woe betide you, if you are the poor patient caught in this vortex, you might feel your welfare is the least important consideration for all these worthies battling it out with their various arguments, ostensibly to protect your interests.*

The dilemma is further compounded by writings and opinions on covert treatment; each adding its intelligent bit to the pool of knowledge, though not always reducing the confusion.

Hence the question still remains: Should he or shouldn't he?

### I.2. A Caveat and Where is the Problem?

Before proceeding further, let me add a caveat. The heading is covert *treatment*, not covert *prescribing*. (Neither is it surreptitious treatment or prescribing, and I shall discuss later why). While prescribing is no doubt a major activity, also important is the counseling of caregivers to help them understand and empathize with the patient's condition. The most important realization that must sink in is that the patient does what he does not to harass or harm others, but because he is sick. Just as a patient of typhoid gets fever, a patient with an uncontrolled psychosis has disorganized behaviour. Just as a patient of typhoid does not *get* the fever to *harass* relatives, similarly a patient of a psychosis does not *get* aggressive or assaultive to *harass* relatives – it is only a symptom of his sickness. Just as symptoms of typhoid abate with treatment, so also the symptoms of a psychosis reduce with treatment. This simple analogy and clear talk often helps caregivers cope better with the patient's disorganized behaviour and decreases the interpersonal stress between them. It also helps boost the morale of the caregiver, which is likely to be very low since he has had to watch the personality disintegration and face the disruptive behaviour of the uncooperative psychotic patient day in and day out. As covert treatment begins to give results and the patient calms down, the caregiver becomes a willing (and, more important, enthusiastic) ally in the treatment programme, not just then but even later. This ultimately helps the patient to recover from his disorder faster and with least distress to all concerned.

Where, then, is the problem? The problem is in the attitude toward covert treatment. It reminds you of a prude's attitude towards sex: every one does it but no one wants to acknowledge it, or even talk about it. There is little systematic research on the use of covert treatment, but there is relatively widespread use (Treloar *et al.*, 2000; Welsh and Deahl, 2002), although it is formally prohibited in all but the most exceptional circumstances.

Whenever there is prohibition of a widely felt need in medicine, at least four different situations may arise with regard to the activity:

1. *Secretiveness and its associated problems*: It is practiced, but clandestinely. Since it is practiced clandestinely, there is scope for misuse, overuse, and other forms of malafide use.
2. *Qualified practitioners may dissociate*: Caregivers may land up with quacks, faith-healers, charlatans, etc. and qualified people may dissociate themselves from it.
3. *Under-reporting*: It is under-reported, if reported at all, due to fear of professional censure.
4. *Scarce standard literature*: It cannot enter into standard biomedical literature.

This is what happened with abortion before it got legalized. It is happening with covert treatment today since it remains a gray area in clinical psychiatric practice and law. It is poorly described in psychiatric literature but is probably more common than one can imagine (Treloar, Beats and Philpot, 2000; Whitty and Devitt, 2005). You hardly find a mention, leave alone discussion, of covert treatment/surreptitious prescribing in any standard textbook of psychiatry. However, one-third (38%) of psychiatrists admitted to using covert treatment in one study (Valmana and Rutherford, 1997), and this figure is likely to be a vast underestimate because the respondents obviously felt uncomfortable accepting on direct questioning that they “deceived” their patients. Moreover, fear of professional censure results in minimal discussion or recording of covert treatment in patients’ case notes, which serves to compound the atmosphere of secrecy and suspicion (Kellet, 1996; Welsh and Deahl, 2002; Whitty and Devitt, 2005).

### I.3. Ethical Issues Involved: Autonomy, Power, Secrecy

The practice is controversial since there are a number of ethical issues connected with autonomy, power, confidentiality, breach of trust and secrecy involved in covert treatment. Let us examine the charges made by those who oppose it and the likely defense.

### I.3.1. Autonomy

*The Charge:* There is the charge that it is overly paternalistic: caregivers cannot take upon themselves the right to decide for a patient. More importantly, there is also the charge that it restricts autonomy: the caregiver's action impinges on the patient's freedom to decide for himself.

*The Defense*: To this there is an equally spirited defense that, rather than impinge on autonomy, it in fact restores it: caregivers actually help the patient get into a proper mental condition to decide for himself. “Ethical, legal and clinical considerations become more complex when the mental incapacity is temporary and when the medication actually serves to restore autonomy” (Wong, Poon and Hui, 2005).

### I.3.2. Power, breach of trust/confidentiality, damage to therapeutic relationship

*The Charge:* The charge is also made that when caregivers engage the services of psychiatry in covert treatment, it amounts to misuse of psychiatrists’ power over unsuspecting patients. It is a breach of trust of the doctor-patient relationship since the relative becomes an intermediary between the doctor and the patient. It is a breach of confidentiality since the problems of a patient cannot be disclosed to anyone else without his knowledge; here it is: so what if he is a relative? It is damaging to the therapeutic relationship that cannot but be based on trust and confidentiality between patient and psychiatrist, both fatally breached in this case. Moreover, it results in deprivation of the rights of the patient to decide for himself.

*The Defense*: To this, too, there is the equally strong defense that withholding necessary medication may actually amount to deprivation of a patient's rights to get well. To protect this right, it is justified that medication be administered, even if it is done covertly. “Although some may view surreptitious prescribing as a deprivation of the rights of the patient, it is also worth remembering that, paradoxically, withholding the medication necessary to effectively treat mental illnesses could also be viewed as a deprivation of the patient's rights” (Whitty and Devitt, 2005) – the right to receive treatment and to get well, even when patients are not able to choose such treatment for themselves.

### I.3.3. Secrecy

*The Charge:* The veil of secrecy connected with covert treatment compounds the suspicion of malafide intent aroused in ethically conscious observers. “Even if, as most carers and some authorities believe, covert medication can be justified, the poor recording and secrecy surrounding the practice in institutions are cause for concern” (Treloar, Beats and Philpot, 2000).

*The Defense:* How can there be recording and lack of secrecy unless the need for such a process is accepted and established in standard scientific literature? Till such time, a perfectly legitimate need may be viewed with suspicion and censored. Witness, for example, the brouhaha raised over the covert treatment of a patient who actually accepted that it helped him, but which resulted in an enquiry committee against the prescribing psychiatrist and suspension of the nurse! (Kellet, 1996). This, in spite of the fact that the treatment was not found to be unethical (Griffith and Bell, 1996). Witness also, for example, that the investigation reported in its aftermath found that over a third of psychiatrists had given drugs “surreptitiously” or lied about giving a drug (Valmana and Rutherford, 1997). This is what follows on suppressing a genuine need.

When there is a veil of secrecy around something, aided and abetted no end by vociferous opposition to the procedure, the result is one of two: 1) either the activity goes underground, like alcohol did during prohibition; or 2) it is reluctantly discarded – although most who used it knew it was needed – like contraception/abortion in a conservative catholic set-up.

Covert treatment has the dubious distinction of suffering both such secrecy and disapproval.

The need of the hour is to explore its need and justification in greater detail, lay clear and firm parameters for legitimate use, follow it up with standard literature and, finally, to establish clinical practice guidelines.

### I.4. Where Found Justified, Where Not

The vacillating attitude of clinicians, researchers and opinion makers does not help either. Most accept the case for covert treatment in those who cannot give consent, like patients of dementia or learning disability (Chua, Choy and Wong, 2001) or those with “severe” dementia or “profound” learning disability (College, 2004; see also Treloar, Philpot and Beats, 2001; Treloar, Beck and Paton, 2001; Øyvind and Knut, 2005). Some also prohibit its use in schizophrenia (College, 2004). Others believe it amounts to winning a battle but losing a war (Levin, 2005). Still others are absolutely prohibitive of this practice (Ahern and Van Tosh, 2005), warning against “the irreversible damage caused by surreptitious prescribing.”

Some others are more accommodative of the practice in psychotic disorders:

Treatment for those who refuse treatment yet who retain capacity can be authorized by statute under Part 5 of the Mental Health Act 1983, whereas treatment for those who lack capacity may be prescribed in their best interests under the common law doctrine of necessity and thus necessary to save life or prevent deterioration or ensure an improvement in the patient's physical or mental health (Department of Health & Welsh Office, 1999; as quoted in Welsh and Deahl, 2002).

Although written in the context of dementia, this is also applicable to psychotic disorders, where the patient is uncooperative, when it talks of “treatment for those who refuse treatment yet who retain capacity.” It is interesting in this connection that caregivers of dementia did not differentiate between medication for a psychiatric disorder and a physical one (Treloar, Beats and Philpot, 2000), meaning thereby that a psychiatric disorder for them was equally a medical problem; neither did the professionals who treated cases of dementia (Treloar, Philpot and Beats, 2001).

Others specially see some justification for its use in psychiatric conditions where there is a documented history of recurrent relapses secondary to noncompliance with medication (Whitty and Devitt, 2005). Some others find justification for the Mental Health Ordinance (which became Law in Hong Kong in Feb. 1999) that “enshrines the principle that adults who are incapable of understanding the general nature and effect of treatment should not be deprived of treatment” (Chua, Choy and Wong, 2001). They also stress that “to bring psychiatric and medical treatment under the same legislation makes sense, since they are similar ethically, given that the aim is to relieve distress and improve health (Treloar, Philpot and Beats, 2001)” (Chua, Choy and Wong, 2001; parenthesis added). This means that they find justification for covert treatment in psychiatry too, since the basic aim – like of all medical treatment – is to relieve distress and promote health.

Having considered some of the contentious issues, let us look at a few practical problems psychiatrists have to grapple with in their day-to-day clinical practice. In Section II, we will then see the gist of the proponents’ and opponents’ arguments and later arrive at a resolution that is practically useful.

### I.5. Outlining Some Familiar Scenarios

Consider the all-too-familiar scenarios outlined below:

### I.5.1. Danger of relapse

A patient was earlier taking medication and was well. He now refuses to take medication and the psychiatrist knows he is in danger of a relapse. Should such a person be administered medication against his will?

### I.5.2. Denial of sickness

Another, previously untreated, patient refuses to accept that he is sick. He moves about suspicious and hallucinating or locks himself up in his room, refusing any outside interaction, even food. Should such a person be administered medication covertly so that he gets calm enough to be finally brought to a psychiatrist?

### I.5.3. Denial of sickness; relatives want to avoid involuntary commitment to institution

An exhausted relative finally approaches a psychiatrist, requesting treatment of a patient who is creating all sorts of problems at home and with the people around. He is reportedly distressed and frightened, tends to hit people, talks/laughs/cries to self, has stopped work and interaction with others and is restless and sleepless. He refuses to accept he is sick, while all around know he is. He cannot be brought for treatment because he just will not come. The relatives do not want to commit him involuntarily to an institution. Should such a relative be helped by suggesting medication that could be covertly administered so that the patient becomes manageable enough to be taken to a psychiatrist?

### I.5.4. Refusal to visit psychiatrist for follow-up

A relative finds the patient better with covertly administered medication. His hallucinations disappear and his delusions are under control. He goes back to work and stops being suspicious of people around. But he refuses to visit a psychiatrist. The relative cannot force the issue on him since the patient threatens to stop treatment if forced. Should the psychiatrist continue administering drugs to such a patient even if he has never seen him?

### I.5.5. Revealing covert treatment

A patient was administered medication covertly and feels well enough to finally come to a psychiatrist. But the relatives have not told him about the covert medication. Should such a patient be told about it? How and by whom?

### I.5.6. Revealing covert ECTs

A patient was admitted involuntarily in a private psychiatric set-up and ECTs had to be administered with the caregivers’ consent. The patient is now well but has amnesia for the episode of hospitalization. Should he be told about the covertly administered ECTs? Whether he demands to know or not?

### I.5.7. Administering covert ECTs

Another patient is given medication but is not improving; in the judgment of the psychiatrist the patient needs ECTs. The patient is unwilling though the relatives are willing. Should the psychiatrist go ahead with ECTs covertly? Should such a patient be informed later, when he gets well, that such a decision had to be taken?

All these are apparently vexing issues. All involve the dilemma connected with psychiatric treatment of an uncooperative patient.

In the next section, let us see further the essential arguments of both sides and then, hopefully, try to arrive at a resolution of the issues.

## II. The Proponents’ Argument, The Opponents’ Argument, -Practical Objections to Covert Treatment, The Resolution

### II.1. The Proponents’ Argument

The common argument of those who support the use of covert treatment is that it is the least distressing method of treating someone who will otherwise come to harm. This is mostly put alongside a pragmatic argument that, as a result of dementia or a learning disability, the patient cannot learn new things and therefore will not be able to learn that the medication must indeed be taken. The other argument is the need for such a procedure to solve practical clinical problems. It has a number of potential advantages in treating patients suffering from severe mental illness (Whitty and Dewitt, 2005). Delay in treating patients with acute psychiatric disorders is associated both with clinical risks and substantial costs (Kelly *et al.*, 2002). The clinical risks are suicide, self-injury, assault, homicide, etc. The costs are due to more prolonged and costlier treatment procedures, hospitalization, psychosocial morbidity and distress, unemployment, increased burden of care, etc. Delaying psychiatric treatment in such patients is connected with “increased morbidity and poorer outcomes in terms of prolonged individual suffering, increased risk of self-destructive behavior, deterioration of the therapeutic alliance and increased physical assaults by the patient” (Whitty and Dewitt, 2005).

### II.1.1. Deleterious effects of untreated psychosis

There is a substantial group of psychiatric patients who either refuse to take treatment or refuse to accept that they are patients. And yet, the relatives and the treating doctor are convinced that they are sick. Untreated psychosis has deleterious effects, which have been well documented (see, for example, Loebel *et al.*, 1992; Norman and Malla, 2001; Perkins *et al.*, 2005; Clarke *et al.*, 2006; Singh, 2007b). Moreover, being ambulatory, they cause significant distress to others around, while remaining bereft of the benefits of modern psychopharmacological treatment. A number of clinicians who have helped countless such patients will vouch for this, as will their distressed families and caregivers, who have thereby been relieved from psychomorbidity due to the patient's intransigence. It also reduces the need for restraint, seclusion and forcibly administered injections (Whitty and Dewitt, 2005), all unpleasant procedures that caregivers maybe compelled to resort to and harbour a great guilt about.

### II.1.2. Reducing caregivers’ burden and empowering them

The problems faced by caregivers of patients with psychotic disorders has received attention. It is they who look after patients and taking care of their problems ultimately helps in taking care of the patients themselves. Covert treatment is one important way caregivers’ burden can be reduced and their problems resolved. The caring role can affect the health and well-being of a carer of a person who has a mental or physical disorder (Cormac and Tihanyi, 2006). Reducing his burden is essential, so that he is available to look after the patient. When caregivers of patients with bipolar illness experience a high burden, the treatment outcome is adversely affected (Perlick *et al.*, 2004). Meta-analyses of schizophrenia studies have demonstrated that family interventions result in reduced burden and increased medication adherence (Pilling *et al.*, 2002; Cuijpers, 1999). Hence, proper sensitization of family members to what is schizophrenia and empowering them to look after such patients goes a long way in controlling the sickness and reduces consequent social morbidity. Part of the process of such empowering and sensitization is to help them by using covert treatment in an uncooperative patient. It greatly helps the therapeutic process to be initiated later. It helps patients get well enough to be taken for treatment. Equally important, it reduces the chances of untreated psychotic patients roaming about at large and becoming a menace to others, hurting caregivers, or locking themselves up and hurting themselves.

### II.1.3. Covert treatment preferable to restraint

In dementia, where the cognitive decline results in forgetfulness to take medication, there are only two options available: restraint and/or covert medication. Restraint is often considered a cruel substitute for covert treatment (Treolar, Philpot and Beats, 2001). Restraint, similarly, is often the last resort of a helpless relative of an acutely psychotic patient who refuses treatment. [Of course, in severe dementia, restraint now will not reduce the likelihood of restraint at times of subsequent dosages.] Covert treatment is preferable to restraint, or to no treatment at all, because it reduces psychomorbidity all round. The helpless relative becomes an empowered relative and often a powerful ally, in the subsequent treatment plan. There is sufficient evidence to show that greater involvement of relatives is beneficial in schizophrenia (Sellwood *et al.*, 2003; Pilling *et al.*, 2002) and starting covert treatment is sometimes the first important way it manifests.

### II.1.4. Earns gratitude of relatives, prevents demoralisation of treating staff

The gratitude of relatives and of the patients themselves as they finally come and accept further treatment (which they often do) – which gives them the best chance to become symptom free – all this motivates the treating psychiatrist to carry on with the procedure, regardless of the cacophony, albeit well-intentioned, of legal hassles and ethical considerations.

Covert treatment in acute psychiatric disorders, moreover, can prevent demoralization of the treating staff since it prevents delay in starting treatment. It can also prevent relapse and the whole rigamarole of hospitalization, certification, etc, as well as the “redirection of limited clinical resources to nontherapeutic activities” (Whitty and Dewitt, 2005).

### II.2. The Opponents’ Argument

### II.2.1. The ethical objection

The argument of those who oppose this is commonly based on ethical and legal grounds. No one has the right to impose treatment on another; no one can connive with so-called caregivers and force conformity on dissenters; no one can abrogate to himself the right to decide for another what is proper or not and force such issues in connivance with relatives on an unsuspecting individual conveniently labeled a “patient.” Legally, too, one is bound not to diagnose or treat in absentia or without the consent of the treated individual. Moreover, cases of overt or covert complicity between relatives and mental health specialists may work to label individuals as patients and deprive them of their freedom of movement. Such labels may also help malafide relatives usurp the property of so-called “patients,” or secure divorce or the custody of a child, etc. – activities with which mental health specialists may overtly or covertly connive.

### II.2.2. The malafide intent and legal hassles

However great may be the noble intention of the treating physician in helping an unknown, and unseen, uncooperative patient and his distressed relative, there is no way such a physician can really know whether the person to whom the covert treatment is being given is really a patient. It may be that a smart relative is trying to lace someone's food or drink to serve his own nefarious ends. In fact, if the clinician really cares for his patient, he should insist all the more that the patient be seen and properly diagnosed and treated. Only then is his beneficent role really and truly played.

Also, if such a case lands up in court, the clinician concerned can find himself in a host of legal tangles, wherein his talk of beneficence may or may not cut much ice with the custodians of the law.

Hence, clinicians are well advised to be pragmatic enough to steer clear of such cases and allow the due process of law, as prescribed for an uncooperative patient, take its course. Also they must firmly resist the urge to act paternalistic (an honest intention which, here, may be problematic if not altogether unjustified) or malafide (a dishonest intention, which is always problematic).

### II.3. Practical Reasons Why Covert Treatment Is Objectionable

There are also some more practical reasons why this practice is objectionable. At least four come to mind here:

### II.3.1. Prevents insight

It may prevent the patient from gaining insight into his problems. “In some cases, insight improves only after recurrent relapses, with the realization by the patient of the relationship between nonadherence and relapse” (Whitty and Dewitt, 2005).

### II.3.2. Encourages denial

Also, it may promote denial of the illness and discourage patients/caregivers from availing of the full treatment. “Surreptitious prescribing may serve to reinforce the patient's view that illness is not present and that he or she does not require treatment” (Whitty and Dewitt, 2005).

### II.3.3. Promotes unhealthy practices in staff

It may also promote unhealthy practices amongst clinical staff as it may become “a cheap means of managing inadequate staff levels” (Whitty and Dewitt, 2005).

### II.3.4. Prevents understanding of noncompliance

Covert treatment may prevent understanding of, and research into, why patients become noncompliant in the first place. In noncompliance many factors are involved: “Patient, doctor, medication and illness factors are associated with poor compliance” (Whitty and Dewitt, 2005). The goal should be to understand these factors so we can “address these reasons before resorting to surreptitious prescribing” (ibid) and thus preempt its very need at some time in the future.

### II.4. The Resolution

What, then, do we do?

As is commonly done with such contentious issues, let us first of all stop being judgmental or close our minds to a fresh approach. Only then can we arrive at a possible solution.

Let us first of all accept certain basic premises and see where it leads us:

### II.4.1. There are psychiatrically sick who need treatment but deny sickness

Whether we like to accept it or not, there is a body of individuals who are moving about in society or who lock themselves up in their homes, but refuse to accept they are sick, although they are.

### II.4.2. Tentative diagnosis in absentia possible

It is possible to make a reasonable tentative diagnosis *in absentia* and to suggest treatment, based on the history described by an observant relative.

### II.4.3. Danger of hurt to self and others

Such individuals cause significant distress to themselves and others, run the risk of suicide and other forms of self-hurt, and may assault others or even commit homicide.

### II.4.4. Psychopharmacology helps

Modern psychopharmacological treatment can help control their disorder and make them productive and less distressing members of society.

### II.4.5. Covert treatment helps restore partial insight

Often, such patients feel well enough with covert treatment to realize that something was wrong with them earlier and so they land up with the treating physician.

### II.4.6. Cautious of malafide intent

The treating physician must, however, beware of conniving, overtly or covertly, with wrongdoing in the name of treatment. Processes must be in place so that he and the patient's relatives cannot do so. [See Point II.4.11.] At the slightest suspicion of being used in such a process, the clinician must insist on seeing the “patient” and confirm that he is, indeed, a patient; otherwise, the physician must refuse to comply with the demands of the relative.

### II.4.7. Covert treatment only a stop-gap

The treating psychiatrist must, moreover, insist on seeing the patient as soon as possible. He must make a note on the case-paper: “To bring patient” and insist that the relatives make sincere attempts to do so. He must not allow covert treatment to continue beyond a reasonable period of time, which may be a few days to a few weeks. In any case, he must not allow this to become a convenient mechanism to continue to treat an uncooperative patient *inabsentia*. (See also point II.4.12 and II.4.13.) The goal should be to get the patient to the clinic to get properly diagnosed and treated after a full examination. Covert treatment is only a stop-gap to help the relative tide over a crisis and to help make the uncooperative patient sufficiently compliant to be brought for treatment.

### II.4.8. How long to give covert treatment, danger of relapse on stopping; will refuse further treatment on disclosure; undermine trust in health care system

In point II.4.7, I mentioned that covert treatment should not continue beyond a reasonable period of time. The following questions can be legitimately raised: How is this to be determined? If covert treatment is stopped once the patient is better, a relapse will result. However, there is no guarantee that the patient will agree to continue with treatment that was covertly given. Firstly, the patient will see no need for this, since he/she is better already and, secondly, to disclose that covert treatment was given by the doctor based on the relatives’ report will undermine the patient's trust in the health care system, may adversely affect family relationships and further impair the patient's compliance.

### II.4.9. Answers to questions in point II.4.8

A “reasonable period of time” means till the patient can come for treatment, which is something the caregiver has to work out. Covert treatment is a stop-gap, not the mainstay of psychiatric treatment. Yes, relapse can occur when covert treatment stops and this is all the more reason why the caregiver should be encouraged to get the patient into regular treatment as soon as possible. There is no guarantee the patient will continue with treatment that was covertly given once he knows it was administered covertly. This situation needs to be handled discreetly. If the bonafide intent is made clear to the patient, if he regains even partial insight and understands how he was before and how he has changed after treatment and if a relative whom the patient trusts and values is involved in the covert treatment process (and he is the one who explains why covert treatment was needed), the problem can be solved to a large extent. To the argument that the patient will not continue with treatment since he is better already, the answer is: on the contrary, it will be the opposite. If he can be made to understand what his condition was before treatment and how it has improved after treatment and the explanation is given in as truthful and convincing a manner as possible, there may be some initial reservations, but the patient will ultimately get convinced – provided he has been adequately treated before this explanation is given. Thus, rather that losing respect for the health care delivery system or suspecting his relatives and impairing compliance, it will strengthen the patient's respect for a system that dares to care and for relatives who ran the risk of censure in their effort to do their best for the patient. Ultimately, the patient will realize that it was done keeping his welfare in mind. He may be suspicious or angry initially, but will relent if: 1) the procedure was carried out in his interest; and 2) if the procedure has been effective in restoring at least partial insight.

### II.4.10. Covert treatment may not help regain insight in all

The charge can also be made that it is too idealistic to imagine that all patients will gain insight and become compliant after the use of covert medication as a stop-gap. Possibly so. Not all patients may gain insight and become compliant but even if a majority of them do, is that not a significant achievement? Noncompliance and a concomitant lack of insight are two of the major problems in psychiatric nonresponders. With so many patients refusing to accept they are sick and relatives afraid to take up the caring role, is it any wonder that noncompliance remains a major problem and that psychiatric morbidity does not decrease as rapidly and efficiently as it should? Even if the majority of such patients can be controlled to become compliant enough to be taken for treatment, it would be a major step forward for the cause of the mental health movement and for reducing social psychomorbidity.

### II.4.11. Preconditions to covert treatment; better safe than sorry

As a measure of abundant caution and for his own protection, the treating psychiatrist must record the statement of the visiting relative on paper and make the relative sign it. Better still, he should make an audio/video recording. If possible, he must have a second relative confirm the statements of the first. If necessary, he may insist that the relative also visit another psychiatrist, give a detailed history and confirm that the other psychiatrist seconds his tentative diagnosis and therapeutic plan. Also, the treating psychiatrist may insist that a known person stand surety or guarantor to justify the intervention and to prevent malpractice by so-called caregivers, similar to what is done when a person opens a bank account or when a person seeks to secure a bank loan. In the event the clinician is pulled up for malpractice, such an individual must agree to testify as to the bonafide intentions of the treating physician.

### II.4.12. Longer covert treatment

In the rarest of the rare case, the psychiatrist may decide to continue with covert treatment for a long time even without seeing the patient or even after seeing one much earlier, if he is convinced the patient needs it but is unwilling to accept medication or enter regular psychiatric care. Continuous monitoring of the patient's state for evidence of therapeutic benefit, as well as to avoid side-effects, is necessary. An alert and concerned relative, who is suitably primed and motivated to care, is a must in such procedures. The precaution suggested in Step II.4.11 maybe fruitfully followed to prevent legal hassles later.

### II.4.13. Covert treatment for the whole duration of therapy

Some patients are reported better with such medication, but they may still refuse to enter a psychiatrist's clinic. Here, the clinician may have to continue with treatment *in absentia* for a reasonable period of time, all the while trying to motivate the relative to convince the patient to come to the clinic for treatment. Very rarely, some patients may have to be continued on such treatment for the whole duration of therapy. The precaution suggested in Step II.4.11 must be carefully enforced, with a periodic reaffirmation in writing, say every three months, by the two relatives and the guarantor.

### II.4.14. Have scientific literature and clinical practice guidelines and get legitimate use legalized

The psychiatrist must unite with other mental health workers and work through their association/s to convince legislative and other authorities of the genuine need for such a therapeutic option. He must, first of all, be convinced he is doing that which is in the patient's interest and neither go overboard nor feel guilty about using such a method on the occasional patient. There must be sufficient scientific literature on covert treatment; it must be brought out of the closet and psychiatry must set in motion the process of establishing clinical practice guidelines by unconflicted authors for its use.

### II.4.15. The unconscious medical patient and the equivalent insight-unconscious psychiatric patient

This will happen only if the psychiatrist finds the process medically and morally justified. Let us take the case of an unconscious patient. No treating physician has any compunctions about restraining an uncooperative, unconscious patient. Such a patient is often tied so that he does not move his hands while intravenous fluids are on; he is administered feeds and medication through a Ryle's tube, without his consent; tubes are inserted through his various body orifices and medicines are administered without his knowledge. Railings are attached to his bed so that he does not fall off. All this, therefore, amounts to covert treatment. No one objects to any of it. *An uncooperative psychotic patient has poor insight and is equally unconscious of the implications of his condition.* He may say that he is not sick, the whole world is, but he is hardly to be believed; his delusions make him say so. What is the proof for this? When he is adequately treated, he develops insight and often accepts that treating him without his knowledge and consent was indeed the only option available. What is the proof that an unconscious medical patient needs to be treating without his consent? The proof is simply that the patient can be diagnosed as sick but he is in no condition to give consent as he is unconscious. Moreover, as the procedure is carried out and such a patient improves, patient and relatives are eternally thankful that the procedure was carried out without waiting for the patient's consent. In fact, waiting for consent in such a situation would be a laughable act. For, unless the patient was administered treatment without his knowledge, he would just not have improved well enough to give further consent. Nobody even considers that an issue. Why should it become an issue in the deluded and uncooperative psychiatric patient? The only difference is that whereas the other patient was unconscious, the psychiatric patient is conscious. But he has no “insight” into his problem. *What is “consciousness” in the medical patient is equivalent to “insight” in the psychiatric patient*. In other words, lack of “consciousness” in the general medical/surgical patient is equivalent to lack of “insight” in the psychiatric patient. If the comatose patient is *unconscious*, the uncooperative psychiatric patient is *insight-unconscious*. Both may need covert treatment to get them well enough to understand what treatment means.

### II.4.16. Covert treatment justified in schizophrenia

There is the argument that covert treatment is unacceptable in schizophrenia: “The covert administration of medication in patients with schizophrenia and other severe mental illnesses where patients can learn and understand that they will be required to take medication is unacceptable” (College, 2004). To this the answer is that covert treatment is unacceptable only if “patients can learn and understand that they will be required to take medication.” If they cannot *learn* or *understand* why they need medication, what then? Patients who are actively psychotic and refuse treatment, who have lost insight, are, at that point in time, patients who cannot learn or understand that they need to take medication. No amount of convincing can make them accept it since they are deluded and the very definition of delusion is that it is a false, unshakeable belief not amenable to reason. The only measure which works is covert medication [or medication under restraint]. Once they are controlled, insight starts returning and patients then accept that they are sick. Often they are grateful that someone had the courage to look after them when they themselves could not understand they were sick. This is what happened, for example, in the case described earlier (Kellet, 1996; Griffith and Bell, 1996). It is a perfect example of justified covert treatment by a psychiatrist and of overenthusiastic officials going overboard in trying to prove they are holier than the holy by making a scapegoat of the psychiatrist and the prescribing nurse.

### II.4.17. Covert treatment justified in schizophrenia (continued)

To carry on from where we left off in the last paragraph: the main reason why some propose its use in dementia and learning disorders but oppose it in schizophrenia is that uncooperative patients of schizophrenia (and related psychoses) are considered those who refuse treatment but retain capacity, while patients of dementia and severe learning disorder lack capacity. This argument needs to be revised because although uncooperative patients of schizophrenia (and related psychoses) apparently retain capacity, it is limited, in fact distorted, since they lack insight. Just as a medically/surgically unconscious patient has to be given covert treatment, similarly an insight-unconscious patient of one of the different psychoses (in the acute phase or otherwise) may also have to be given covert treatment till he regains partial insight. It helps control psychotic symptoms and restores enough insight for the patient to know he needs treatment. The argument against covert treatment also is that people with schizophrenia have the capacity to learn and so can learn by being required to take medications, but if medications are given covertly it may well fuel their paranoia. However, the patient who has lack of insight cannot learn unless he regains that insight and he may need covert treatment to facilitate this process. Covert treatment can fuel the paranoia, true, but it can also control the psychotic symptoms sufficiently to allow regular treatment to be initiated. And in a patient who refuses to accept he is sick and where involuntary commitment is not an option to be considered, covert treatment is the only option, apart from physical restraint. Ultimately, a choice has to be made between a larger beneficence (control of symptoms and start of therapy) and a smaller malevolence (necessary therapy, but without knowledge and consent).

### II.4.18. Patients, and others, who campaign against covert treatment

The patients who campaign against covert treatment are often those: a) who were given covert treatment, but half-heartedly and/or treated incompletely later; b) whose sickness had already become chronic; or c) who were coerced into it with malafide intent. That does not justify our avoidance of it in the proper case. These patients manage to attract the support of human rights activists and some mental health workers, even professional psychiatrists. They rant about its ill effects. More importantly, they cause doubts about its legitimate use even in the rest who should know otherwise.

### II.4.19. Those helped should come out of the closet

Those who have been helped by covert treatment should be encouraged to come out of the closet and speak to a wider audience. Similarly, caregivers who have benefited should speak about it. Those in whom it was suggested but who could not adopt it should also speak about whether it helped or harmed their cause. Those who speak against the process are quite vociferous, while those who have been helped enjoy its benefits and remain quiet. That hardly helps the cause of better patient care. Speaking to a wider audience about the positive contributions of psychiatry is a major task before psychiatry today (Singh, 2007a).

### II.4.20. Covert treatment, not surreptitious prescribing

The psychiatrist administers covert treatment in the best interest of the patient but without his knowledge or consent. That is not the same as surreptitious treatment. The term “surreptitious” has not only a tinge of stealth, it has a strong whiff of malevolence.* The term and such an approach, is to be avoided. The right term and procedure is “covert treatment.” *Covert treatment can be defined as temporary treatment without knowledge and consent, with the bonafide intention of getting the patient well enough to consent and take further treatment*.

### II.4.21. Covert treatment bonafide, surreptitious treatment malafide

Let me put it a little differently. Treatment without knowledge or consent could be bonafide or malafide. We call it covert treatment when the purpose is bonafide and surreptitious treatment when it is malafide. We justify only the bonafide purpose. We call it covert treatment when the purpose is to control an acute phase of psychosis so that an uncooperative patient lands up for treatment, to prevent a relapse in another or to retain control over symptoms in a third, etc. The intention is to care for, comfort, control the patient and, ultimately, to get the patient into regular treatment and hopefully control the psychiatric disorder. Surreptitious cannot combine with bonafide intent. It is, by its very nature, malafide. *The term surreptitious treatment should be reserved for activities such as labeling a person as a patient when he is not one, when somebody is stealthily coerced into conformity just because he is deviant, when psychiatry and psychiatrists are used by self-serving relatives to further their nefarious agenda of usurping other's rights by getting them labeled as patients.*

Covert treatment, within limits and with riders, is acceptable. Surreptitious treatment is never acceptable. The term “covert treatment” is preferable to “surreptitious treatment/prescribing” since the former implies the bonafide intent while the latter emphasizes the malafide action. It is only a bonafide intent that can justify a malafide action, if ever such an action is justifiable. Hence the term surreptitious treatment as a synonym of covert treatment should be dropped. Also, the meaning that it connotes should not become the driving force in treating the uncooperative patient, psychiatric or otherwise. Rather the meaning and intent should all along be to care for the patient and get him symptom-free to lead as productive a life as is possible, as also to get him into legitimate out-patient or in-patient care.

### II.4.22. Distinction between legitimate treatment and labeling

It is important that both the establishment of psychiatry and the activists for human rights, etc, understand the fine distinction between legitimate treatment and labeling. Both can go overboard. Legitimate treatment is when patients are diagnosed according to well-defined criteria and symptom profile, although occasionally *in absentia*, with the intention of helping the patient get well and to enable the relatives to handle them better (an example of covert treatment). Labeling is when the psychiatrist collaborates, knowingly or unknowingly, with relatives who have a malafide intent (an example of surreptitious treatment). To believe all people who are reported as being sick by relatives is as invidious as to believe that all people treated *in absentia* are treated with malafide intent. If proper care is taken (as outlined in points II.4.6, II.4.7 and II.4.11), so much of psychopathology to which society is needlessly exposed in the name of patients’ rights can be reduced and society and patients themselves, benefited. This is obvious to anyone who has treated any such uncooperative patient, got him into regular treatment later and made him well enough to become a productive member of society. I count myself in that category.

### II.4.23. The essence of covert treatment

Hence covert treatment, which is temporary treatment without knowledge and consent, is an essential procedure in the psychiatrist's armamentarium, which he must use very judiciously, in the rarest of rare cases. But he must not hesitate to use it when needed or feel guilty about using it, provided he is firmly convinced it is needed for the welfare of the patient and is the only available option to tide over the crisis for a distressed, but concerned, relative. All the while, the psychiatrist must try to get the concerned patient into regular psychiatric care; he must insist that the procedure be used only as a stop-gap, never as the main form of therapy; he must always be alert to the possibility of malevolence inherent in such a process and keep away from conniving in anything even remotely connected with such a motive in a suspect relative; and must always take due precautions to ensure that he does not land into legal tangles later.

### II.4.24. Do not harm, but also dare to care

Although we must believe in non-malfeasance (above all, do no harm), we must equally believe in the best of beneficence (even above that, dare to care).

### II.4.25. The issue of non-harm as a justification

If harm does not arise from non-treatment then one cannot justify its imposition. Which also means if harm arises from treatment, then one has a duty not to provide it. But if harm arises from non-treatment, then one has a duty to provide treatment to those who cannot choose. This is the rub of the discussion.

### II.4.26. The issue of harm and hurt

While both hurt and harm involve distress to another, harm also involves malevolence in the perpetrator. The intention is the culprit (Singh and Singh, 2006). *Hurt* occurs often while we effect cures and offer care. *Harm* occurs following manipulation and exploitation by caregivers and when unnecessary procedures are carried out (ibid). In the case of covert treatment, hurt is involved: hurt to the patient's right to give informed consent. The psychiatrist must ensure that harm is never involved, for then it becomes “surreptitious” treatment and means conniving against the patient's best interests by usurping his right to refuse treatment which is unnecessarily and stealthily taken away. If the process of resolution suggested here is strictly adhered to, the chances of harm are diminished, if not totally obliterated. Even hurt is minimized.

### II.4.27. Reservations understandable, but alternative unavailable

Some clinicians can have justifiable reservations about prescribing covert treatment to patients whom they have not seen. According to them, if the doctor has not made a first-hand assessment and is prescribing treatment based on relatives’ report, it is dangerous, unethical and illegal. To that, the only answer is to ask whether they have a viable alternative to offer, apart from involuntary commitment? It is easy for the clinician to demand to see the patient but, in practice, the caregiver may find it very difficult to comply with the doctor's wishes when the patient has locked himself up or is menacingly assaultive (and also powerfully built). It is in such cases and only as a stop-gap till the patient can be brought to the clinician, that covert treatment can be justified.

### II.4.28. Proper assessment in person ideal, but interim assessment in absentia -occasionally necessary

The charge can be further made that it is fine to write about “daring to care” but care of the patient includes a proper assessment before appropriate treatment is given. Proper assessment cannot be made *in absentia* and so covert treatment is unjustified. The answer to this is: of course, proper assessment is necessary before appropriate treatment is given. However, proper assessment involves the physical presence of the patient. An interim assessment *in absentia*, which can ensure a tentative diagnosis so that interim treatment can be started in a non-compliant patient, solely with the purpose of making him compliant enough to be brought for regular treatment, is equally justified; especially so when involuntary commitment is not an option. If someone has a better suggestion to offer for ensuring such compliance, he is most welcome to put it forward.

## III. An Advance Directive and Answering Questions Raised in I.5 Earlier

### III.1. An Advance Directive and Health Care Proxy for Covert Psychiatric Care

### III.1.1. What is an advance directive and who is a health care proxy?

Let us first see what an advance directive is and who is a health care proxy. Then I will lay down an advance directive for myself and appoint a health care proxy to implement it; because, charity must begin at home.

“An advance directive for psychiatric care is a legally enforceable document that specifies the manner in which psychiatric treatment decisions are to be made in the event that a person later becomes incompetent to make informed health care decisions” (Gallagher, 1998). “A health care proxy is an advance directive that allows an individual to indicate in writing who can act on his behalf when he lacks the capacity to make health care decisions and what limitations he is placing on this authority. Of great interest in medical settings, health care proxies are beginning to receive more attention in psychiatric settings” (Geller, 2000).

In this connection, it is interesting to note that there is a reasonable literature on advance directives and health care proxies in medical and psychiatric care, which interested readers may browse with profit (Raymark *et al.*, 1995; Gallagher, 1998; Geller, 2000; Widdershoven and Berghmans, 2001; Papageorgiou *et al.*, 2002; Srebnik *et al.*, 2003; Srebnik, Appelbaum and Russo, 2004; Appelbaum, 2004; O'Connell and Stein, 2005; Srebnik *et al.*, 2005; Foti *et al.*, 2005; Swanson *et al.*, 2006). Most of it discusses the pros and cons of advance directives and health care proxies, often used in a medical setting but gaining greater acceptance in psychiatry too.

### III.1.2. Charity begins at home: An advance directive for covert psychiatric -treatment

As a psychiatrist who has handled patients in this manner and can vouch for its effectiveness in the carefully selected case, I have no hesitation in signing this advance directive and health care proxy for covert psychiatric care for my own self:

*III.1.2.1. Consent for covert treatment*:If ever I develop a psychosis and if I were to lose insight and refuse treatment, believing my delusions were the reality while what the world outside thought was delusional; and further if I were a management problem for my caregivers and society, e.g, was actively suicidal, homicidal or harmful to self or others in various other ways, I would like to place on record here and now that I be covertly treated till I become sufficiently compliant to understand what my sickness is, and be able to voluntarily consent to treatment.

*III.1.2.2. Absolving caregivers*: As a corollary to the above, I would absolve my caregivers of any malevolent intent in helping me get rid of my psychosis, provided my properly qualified psychiatrist felt such covert treatment was the only viable alternative in the circumstances.

*III.1.2.3. Consent for therapeutic processes*: As a further corollary to this, I agree that I may be subjected to all reasonable therapeutic psychiatric processes, as far as possible with my consent but if necessary without it, but with the consent of my caregivers wherever possible and of legally appointed guardians where it is not possible, to see to it that my psychosis is controlled and I can come back to as much of sanity as modern psychiatric treatment can provide for at that point in time.

*III.1.2.4. Accepting worth of covert treatment*: Having seen the distress and difficulty caregivers of the uncooperative patient undergo and the benefit that carefully selected limited-period covert treatment can offer them and having seen that such interventions can help the uncooperative insightless patient get back to sanity if not total control of psychosis, I have no hesitation in accepting this as the only proper course for myself if I were ever to become such a case.

*III.1.2.5. Not sanctioning malafide use*: Always, provided my caregivers were honest in their intent and the administering physician was competent in his knowledge.

*III.1.2.6. Health care proxy*: I would not hesitate to appoint a health care proxy, as of now, Mr./Ms ABC (my spouse/parent/friend/lawyer/etc.) as a fit person to take such a decision, after due consultation with Dr. XYZ (treating psychiatrist) and if necessary with a second opinion of Dr. DEF (second psychiatrist).

*III.1.2.7. Informed, free consent*: This consent I give freely, being in full control of my senses, without any force or coercion from any quarter whatsoever.

### III.2. Taking Up Questions Raised At The Beginning [In I.5]

Having discussed the issues involved and resolved them, at least for now, let us try to answer the questions raised in the clinical scenarios presented earlier in section I.5.

### III.2.1. Danger of relapse

Q1. A patient was earlier taking medication and was well but, now, refuses to take medication and the psychiatrist knows he is in danger of a relapse. Should such a person be administered medication against his will?

Ans. 1. Yes; but with the express intention of preventing relapse and getting him into regular inpatient/out-patient care as soon as possible. It must be done with all the precautions suggested in II.4.11.

### III.2.2. Denial of sickness

Q2. Another previously untreated patient refuses to accept he is sick. He moves about suspicious and hallucinating or locks himself up in his room, refusing any outside interaction, even food. Should such a person be administered medication covertly so that he gets calm enough to finally approach a psychiatrist?

Ans. 2. Yes; but with a detailed, recorded history as given by the caregiver, which should be signed by the caregiver as well as another relative. It would be advisable to have a second concurring opinion from another psychiatrist as a precaution (especially if there is even a mild possibility that medicolegal issues may be involved later). In addition, the caregivers should be instructed that they are to bring the patient to the clinic as soon as it becomes possible. If there is even a hint of malafide intent on the part of the caregiver, the psychiatrist must insist on seeing the “patient” immediately. In case he does not take oral feeds, it may be necessary to restrain and give both oral feeds and covert medications. If he does take oral feed but refuses medication, the choice is between covert medication and use of restraint each time medication is to be administered. Covert treatment is preferable when it is not practically possible to apply restraint.

### III.2.3. Denial of sickness, relatives want to avoid involuntary commitment to institution

Q3. An exhausted relative finally comes to a psychiatrist requesting treatment of a patient who is creating all sorts of problems at home and with the people around. He is reportedly distressed and frightened, tends to hit people, talks/laughs/crys to self, has stopped work and interaction with others and is restless and sleepless. He refuses to accept that he is sick while all around know he is. He cannot be brought for treatment because he just will not come. The relatives do not want to commit him involuntarily to an institution. Should such a relative be helped by suggesting medication that could be covertly administered so that the patient becomes manageable enough to be taken to a psychiatrist?

Ans. 3. First reassure the relative that the problem is manageable; rest as in Ans 2.

### III.2.4. Refusal to visit psychiatrist for follow-up

Q4. A relative finds the patient better with covertly administered medication. His hallucinations are gone and his delusions are under control. He goes back to work and stops being suspicious of people around. But the patient refuses to visit a psychiatrist and the relative cannot force him to since the patient says that in that case he will stop treatment. Should the psychiatrist continue administering drugs to such a patient even if he has never seen him?

Ans. 4. Yes. But justified in the rarest of rare cases, even of covert treatment. Always with the insistence that this not be used as an easy option out and always insisting that the caregiver try and get the patient into regular treatment as soon as possible. The help of a relative whom the patient trusts and listens to is very helpful here. [Sometimes extrapyramidal symptoms, akinesia, akathisia unwittingly help].

### III.2.5. Revealing covert treatment

Q5. A patient was administered medication covertly and feels well enough to finally approach a psychiatrist; but the relatives have not told him about the covert medication. Should such a patient be told about it? How and by whom?

Ans. 5. Information imparted, if asked for. Who gives the information depends on the circumstances. It should preferably be the caregiver, if needed with the help of another person whom the patient trusts/respects. Rarely, the psychiatrist may have to be the person to do this. Sometimes, after being told by the caregiver, the patient may also need to confirm whether the treatment was indeed necessary, which the psychiatrist must confirm.

### III.2.6. Revealing covert ECTs

Q6. A patient was admitted involuntarily in a private psychiatric setup and ECTs had been administered with the caregivers’ consent. The patient is now well, but has amnesia for the episode of hospitalization. Should he be told about the covertly administered ECTs? Whether he demands to know or not?

Ans. 6. If the patient is well and does not demand to know, there is no obligation to do so. But in case the patient demands to know the facts, he must be properly informed, with the explanation that it was given in his best interest and was the only worthwhile option available in the circumstances. Of course, before the psychiatrist says this to the patient he must be convinced that it indeed was so.

### III.2.7. Administering covert ECTs

Q7. Another patient is given medication but is not improving satisfactorily and, in the opinion of the psychiatrist, needs ECTs. The patient is unwilling, though the relatives are willing. Should the psychiatrist go ahead with ECTs covertly? Should such a patient be informed later, when he gets well, that such a decision had to be taken?

Ans. 7. Only in the rarest of rare cases: when the psychiatrist is firmly convinced, by research literature and his own clinical experience, that ECTs can definitely be helpful in this case. Additionally, it must only be done after exhausting all other options of therapy and after giving those options reasonable time to act. In the case of an emergency, the legal process for involuntary treatment/hospitalization is a much better option as it helps protect the psychiatrist later. Occasionally, the psychiatrist may have to stick his neck out to help, but discretion is paramount here. The moment he knows there is a potential legal hassle, he must resist the temptation to be a Don Quixote and hand over such a case for legal processes to handle.

## Concluding Remarks

1. What is covert treatment and where is it justified:
  Covert treatment or temporary treatment without the patient's knowledge and consent, is seldom needed or justified. But, where needed, it remains an essential procedure in the psychiatrist's armamentarium, to be carried out cautiously but without guilt or fear of censure. However, he must use it very judiciously, in the rarest of rare cases, provided: i) he is firmly convinced it is needed for the welfare of the patient; ii) it is the only option available to tide over a crisis; iii) he ensures that efforts are on all the time to try and get the patient into regular psychiatric care; iv) he insists on its use only as a stop-gap; v) he is always aware of the chances of malevolence inherent in such a process and keeps away from conniving in, or associating with, anything even remotely connected with it; and vi) he takes due precautions to ensure that he does not land into legal tangles later.
2. Non-malfeasance, but also beneficence:
  Do no harm, true, but also dare to care. The first has no meaning bereft of the second, just as the second has no meaning if it sidelines the first.
3. Psychiatry needs to be more forthright about legitimate therapeutic options, covert treatment being one:
  Psychiatry needs to be a little less circumspect about its procedures and also a little less apologetic about its processes. For that, it will have to be sure of its role as a premier agency to treat the mentally sick and also reduce the burden of distressed caregivers, as well as to reduce the resultant social psychopathology. Covert treatment is one such procedure which can help in selected cases.
4. Safety first, but firm action equally important:
  It is of course good to be cautious, and better to be safe. But, it is unpardonable to be vacillating and suicidal to be paralyzed into inaction.
5. Use term “covert treatment”, drop use of term “surreptitious treatment/prescribing”:
  The term “covert treatment” is preferable to “surreptitious treatment/prescribing”, since the former stresses the bonafide intent while the latter emphasizes the malafide action. It is only a bonafide intent that can justify a malafide action, if ever such an action is justifiable. Also we must drop use of the term surreptitious treatment as a synonym for covert treatment and use the former only when the intent is malafide.
6. Establish Clinical Practice Guidelines for Covert Treatment
  The need of the hour is to explore the need and justification for covert treatment in greater detail, lay clear and firm parameters for legitimate use, follow it up with standard literature and, finally, establish clinical practice guidelines by unconflicted authors.

### Take Home Message

1. Covert treatment is seldom needed or justified.
2. Where necessary, it must be carried out without guilt or fear of censure, only for the welfare of the patient and never with intent to cause harm and with due precautions that one does not land into legal tangles later.
3. The term “covert treatment” is preferable to “surreptitious treatment/prescribing.” The former is to be used when the intent is bonafide, the latter when it is malafide.
4. The need is to have standard literature on covert treatment and to ultimately establish clinical practice guidelines by unconflicted authors.

## Questions That This Paper Raises

1. Can we visualize a situation when compliance with psychiatric treatment will make covert treatment redundant?
2. Ethical concerns and activism are often at loggerheads with establishment concerns. This is universal and applies to psychiatry as well. It appears necessary to curb misuse of power and malfeasance. Can we visualize a situation when ethicists, activists and establishment psychiatry will work in tandem to ensure beneficence and patient welfare?
3. What are the dangers and pitfalls of covert treatment that may override any advantages it may confer in psychiatry and in the promotion of mental health?
4. Are activists and ethicists liable to the charge that they take up theoretical issues and make tangential arguments as they are oblivious of ground realities? And that they are likely to go overboard in their objections to genuine needs?
5. What would those who object to covert treatment do if they had to manage a close family member who was actively psychotic and refusing treatment or locking himself up? Would they only think of involuntary commitment and/or admission in a mental hospital or would they think of covert treatment as an alternative before resorting to more drastic means?
6. How would those who object to covert treatment manage a close family member who stops treatment and suffers relapse and is not ready to undergo treatment in a clinic/hospital? Or stops treatment and is in danger of a relapse? Would they think of covert treatment as a therapeutic option?

## About the Author

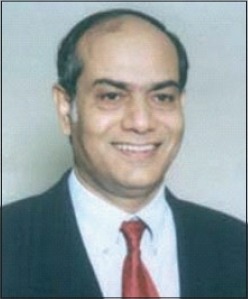



Ajai R. Singh M.D. is a Psychiatrist and Editor, Mens Sana Monographs, (http://www.msmonographs.org). He has written extensively on issues related to psychiatry, philosophy, bioethical issues, medicine and the pharmaceutical industry. He has also written a book on music and a book of poems (awaiting publication).

## Footnotes

* Perhaps the poor patient may develop confidence that each person making decisions around him is acting in his welfare, and for imparting him the best care possible, if these agencies’ actions become synergistic, rather than contradictory, as they are today.

* Covert means ‘secret’ or ‘disguised’; surreptitious means ‘underhand’, ‘done by stealth’ (Sykes, 1985). The former is neutral as to intent, the latter is strongly negative.
